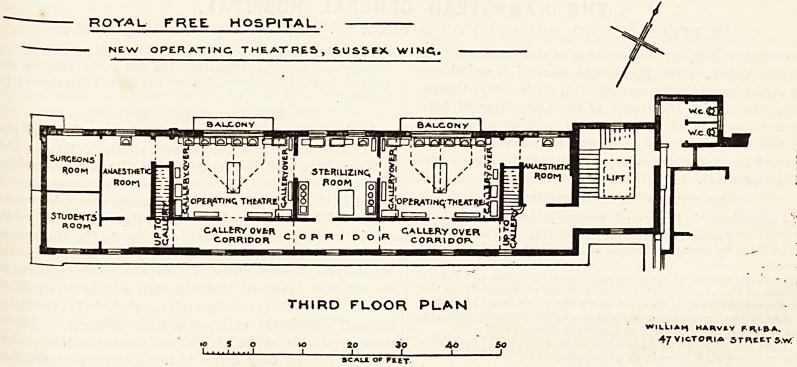# The Royal Free Hospital

**Published:** 1907-12-07

**Authors:** 


### HOSPITAL ADMINISTRATION.

### CONSTRUCTION AND ECONOMICS.

### THE ROYAL FREE HOSPITAL.

THE NEW OPERATING THEATRES.

ON Tuesday afternoon, December 3, her Royal Highness Princess Christian of Schleswig-Holstein, as President of the Royal Free Hospital, opened the new operating theatres which have lately been completed. The new mortuary was dedicated also on the same occasion.

When it is mentioned that the only hitherto existing theatre in the hospital has no anæsthetising room, and no connection with some of the surgical wards other than across the central (uncovered) quadrangle of the buildings, it will be realised how pressing the other needs of the hospital have been which have prevented until this year the construction of the new theatres. These are two in number, theatres. The lift is at the corner where the north and east blocks meet, and all the surgical wards are now situated in one or other of these two.

The arrangement of the theatres and annexes is, as will be seen by reference to the plan, a somewhat straggling one; but this is unavoidable owing to the long, narrow shape of the substructure. In the centre is a large sterilising room containing a high-pressure steam steriliser, and boilers from which sterile water, salt solution, etc., are delivered by pipes through the walls into the theatres. It is almost superfluous to say that everything is designed to hinder the accumulation of dust and to facilitate its removal. Not only are the floors of the two theatres laid on a slope, so that they may be flushed, but all

ROYAL FREE HOSPITAL.

NEW OPERATING THEATRES, SUSSEX WING.

THIRD FLOOR PLAN

WILLIAM HARVEY F.R.I.B.A.

47 VICTORIA STREET S.W.

and space for them has been found only by creating a new storey on the roof of the north block. From this lofty eminence the windows of the theatres, which face approximately N.N.W., command an extensive prospect over the small property lying towards King's Cross. These very large windows, continued into a top light, must undoubtedly allow a splendid illumination of the field of operation; whether they will turn out to be the cause of undue warmth in summer and of the reverse in winter, experience alone can decide. It should be mentioned that certain portions can be opened if necessary, and smut-strainers substituted.

The structure of the north block having been, after some slight misgivings, pronounced capable of bearing these additions, it has been necessary to strengthen very considerably the ceiling of the old top storey. This, the floor of the new theatres, is now of fireproof concrete, finished in terrazzo. Another alteration which has been necessitated in the interior economy of the hospital is the interchanging of some of the medical and surgical wards, so as to minimise the distance of the latter from the switches and similar fittings are cased in, so as to be protected from damage when the walls, windows, or ceilings are sluiced down with a hose. The hot-water radiators, six in each theatre, swing out from the walls for the same purpose. Whether it will prove practicable to carry out this sluicing thoroughly without damaging anything remains to be seen: the experiment is at least worth watching.

The provision of accommodation for students is always a difficult matter in operating theatre construction. For all practical purposes it is only the assistants who can ever really see the details of an operation, though hospital architects still provide elaborate and costly tiers of seats. At the Royal Free Hospital a praiseworthy attempt has been made to meet this difficulty, though its success is not likely to be more than partial. Along three sides of each theatre runs a marble gallery, quite high up near the roof. This gives all the occupants, at least all those next the rails, an equal view of a centrally-placed operating table; and the high elevation may possibly enable them to see over the shoulders of those around the table. For such operations as can be observed at a distance this gallery will answer well; the difficulty of arranging for those which cannot be so watched is one which none of the ingenious complications of overhead mirrors or any other device has yet succeeded in solving.

One more point which strikes the eye is the rather unnecessary profusion of brass work, which it is a great labour to keep bright. The staff of the theatres is to consist of a sister, two nurses, and a man for the instruments; and this number is not too many for the work that will be done. In consequence, all unnecessary labour should be as much as possible dispensed with.

In the corridor which runs along the south side of the rooms it is proposed to set up cupboards for the stock of theatre linen, towels, etc. This corridor is not a very wide one now, and along it patients will be wheeled to the far end in order to reach the second anæsthetising room. It will be a distinct disadvantage to encroach on this space by cupboards, and possibly the room now assigned to the students may have to be expropriated for the purposes of a store-room.

It is contemplated that one of these theatres should be reserved for septic cases as far as practicable; it is also proposed to utilise the anæsthetising rooms as accessory theatres for minor operations of special departments, such as the dental, aural, and so on. The old theatre on the ground floor of the east block will still be made use of for out-patient operations; and the room next it, which was formerly curtained off into an electro-therapeutic part and a waiting-room for patients about to be anæsthetised, is now to be handed over entirely to the electricians, and cannot fail to enhance their activities and opportunities.

In these extremely modern and beautiful theatres the operations, which at this hospital have increased tenfold in twenty years, will in future be conducted with every advantage, and will doubtless continue yearly to increase in number. The work has been carried out under the direction of Messrs. W. Harvey, F.R.I.B.A., and A. T. Snell, A.M.I.C.E.

## Figures and Tables

**Figure f1:**